# Exposing the chameleon-like nature of racism: a multidisciplinary look at critical race theory in higher education

**DOI:** 10.1007/s10734-022-00879-9

**Published:** 2022-05-27

**Authors:** Judith C. P. Lin

**Affiliations:** 1 California State University, Northridge, CA, USA

**Keywords:** Critical race theory, Higher education institutions, Race/racism, Multidisciplinary, Higher education scholars and practitioners

## Abstract

In higher education institutions, critical race theory (CRT) is known to be associated with fields that study racial disparities or systemic oppression such as law, education, and ethnic studies. The impression that CRT is unrelated to fields like business or computer science may have led scholars and practitioners from these disciplines to put their focus on elsewhere than on racial inequality and its implication in their research and practice, despite apparent need. To counter such fallacy, this review article—focusing primarily on the US context—discusses CRT literature in fields where its presence is less known which are nevertheless among the major domains of higher education institutions: health sciences, computer science and information technology, sports, business, and religion. By discussing example research of how scholars have utilized CRT in different fields to challenge the race-neutral thinking that often obscures structural racism, this paper exposes racism’s ability to alter manifestations and to appear through various shapes and forms within the higher education context. Initial recommendations on how educators may engage in further discussions or actions will also be considered. This paper concludes that racist ideologies are often hidden behind discipline-specific vocabulary or technical language, and it is by tackling the ideologies at work underneath the technicalities can we address the chameleon-like nature of racism more effectively.

## Introduction

Critical race theory (CRT) is an intellectual movement that emerges out of law in the 1970s and began to gain currency in the late 1980s and the 1990s in the USA (Bell, 1980; Crenshaw et al., 1995; Delgado & Stefancic, 2017). As a race-centered epistemology and a praxis-oriented framework, CRT offers a lens through which to interrogate how racist ideologies, colorblindness, and White supremacy have shaped social structures, practices, and discourses in society (Adams & Salter, 2011; McMurria, 2016; Yosso, 2002). Today, CRT has spread to other fields of scholarship and around the world.

In higher education institutions, CRT is known to be associated with fields that study racial disparities or systemic oppression such as law (Crenshaw, 2011; Crosby, 2016; Delgado & Stefancic, 1993), education (Ladson-Billings & Tate, 1995; Solórzano, 1998; Vargas et al., 2021), and ethnic studies (Gold, 2016; Rabaka, 2007; Yosso & García, 2007). The impression that CRT is unrelated to fields like business or computer science may have led scholars and practitioners from these disciplines to put their focus on elsewhere than on racial inequality and its implication in their research and practice, despite apparent need. To counter such fallacy, this review article focuses on CRT literature in fields where its presence is less known but which are nevertheless among the major domains of higher education institutions: health sciences, computer science and information technology, sports, business, and religion. This review focuses primarily on the US context. As our discussion will show, racist ideologies are not only well and alive in these fields, but they are often hidden behind discipline-specific vocabulary or technical language. It is only by tackling the ideologies at work underneath the technicalities can we expose and address the chameleon-like nature of racism more effectively. The purpose of this review article is twofold: First, through presenting literature focusing on less-expected higher education spheres, this paper seeks to expose racism’s ability to alter manifestations and appear through various shapes and forms within the higher education context. By doing so, this paper also showcases the breadth of the application of CRT. Second, this paper seeks to raise awareness among higher education scholars and practitioners of CRT’s relevance in various domains of higher education.

This paper is part of a larger project, the CRT Meta Project, where I compiled and indexed research literature around CRT across disciplines from October 2020 to April 2021 (more below). The search on academic databases led to the identification of over 1000 articles around CRT from a wide range of academic disciplines. With the rising popularity of CRT in the last decade, the sheer number of articles found is unsurprising. However, the multidisciplinary nature of the project provides us with a fresh understanding of ways in which CRT has been employed in research in different fields at a meta level. To date, no known scholars have offered a bird’s-eye view of CRT that encompasses discussion from across disciplines. As such, this article contributes to the broader academia by sketching out CRT in less-expected higher education spheres in a single paper.

In the following, I will first discuss the CRT Meta Project and the inclusion and exclusion criteria concerning the use of literature in this paper. I will then consider how CRT has been utilized by scholars to interrogate the role of race and racism at work in structures, practices, and discourses in health sciences, computer science and information technology, sports, business, and religion. At the end of each subsection, I will offer initial recommendations regarding how higher education scholars and practitioners may engage in further discussions or actions as it relates to each field. The paper will conclude with suggestions for future research directions.

## Method

### The CRT meta project

The CRT Meta Project was initiated with the goal to make available sample CRT sources for my institution (located in the USA) with the hope that CRT will be further integrated into the institution’s curriculum. From October 2020 to April 2021, the term “critical race theory” was used to search for articles in over thirty academic databases. It was the CRT Meta Project’s design to be extensive in its construction, thus relying on my institution’s library databases page, I searched databases that are listed under different fields. The databases searched included, for example, GreenFILE, CINAHL Plus with Full Text, ACM Digital Library, PubMed, Cochrane Library, Schomburg Studies on the Black Experience, Chicano Database, PsycINFO, JSTOR, ScienceDirect, SAGE Journals Online, Web of Science, ABI/INFORM Complete, Business Source Premier, ATLA Religion, and Historical Abstracts. The list encompasses disciplines that range from health sciences, STEM (i.e., science, technology, engineering, and mathematics), and social sciences, to business, law, and the humanities.

The initial search was filtered to show articles published between 2001 and 2020/1 to capture the most recent publications. In the case that a search returned too many results (e.g., PubMed) or too few results (e.g., GreenFILE), the date filter was then adjusted to narrow or expand search results. Once articles were identified, references were imported to EndNote to be sorted by discipline based on the programs that my institution offers.

### Inclusion and exclusion criteria concerning the use of literature

Relying on the aforementioned EndNote library and working inductively, literature was selected from health sciences, computer science and information technology, sports, business, and religion for discussion in this paper. The increased importance of praxis-oriented research in higher education (Mahon et al., 2019; Smith et al., 2010) accounts for the selection of the five fields. Over 100 articles were collected in health sciences in EndNote, 27 in computer science and information technology, 45 in sports, 19 in business, and 20 in religion (note that not all articles meet the inclusion criteria as explained below).

The inclusion of literature in this paper is based on the following criteria across all five fields: peer-reviewed articles published between January 2001 and April 2021, authors’ acknowledgement that they engage their discussion through a CRT lens (as opposed to mentioning CRT in passing), and the relevance of the study to higher education (thus excluding studies in K-12 settings). Because my institution is located in the USA where CRT has garnered national attention and been a subject of fierce debate—particularly since President Trump issued an executive order in 2020 which threatened the efforts to address racial disparities in the workplace (subsequently revoked by President Biden in 2021)—this review focuses primarily on the US context to reflect the reality of the immediate milieu. Whenever applicable, the historical development of CRT in a given field was traced by filtering the articles by year in EndNote. Based on the criteria set out, I reviewed all articles that meet the inclusion criteria in all fields but health sciences. Because more than enough articles meet said criteria—particularly from health sciences—literature with a wide variety of research foci were selected to showcase the breadth of the application of CRT. Since it is by design that this paper offers a broad scope for discussion, the literature review will not be comprehensive. It will, however, serve as a broad sample of representative research on CRT to begin a dialogue around the ways that CRT can be a useful lens for many disciplines in higher education institutions to work toward racial equity.

## Come again… critical race theory in what fields?

This section reports the application of CRT in five fields, namely, health sciences, computer science and information technology, sports, business, and religion. Each subsection will start with an overview that considers the state of CRT in each field. Then, I will discuss examples of research where scholars have utilized CRT in their fields to challenge the race-neutral thinking that often obscures the structural racism observed in each field. The discussion is arranged on the basis of similarity in the concepts or themes of interest. Each subsection will end with initial recommendations on how educators may engage in further discussions or actions.

### Health sciences

Though not always readily noticeable, structural racism has long been interwoven in health research, healthcare systems, and health education. Yet while education drew upon legal scholars and CRT as early as the 1990s (Ladson-Billings & Tate, 1995; Solórzano, 1998), a more robust discourse of CRT in health sciences in general and public health in particular did not emerge until two Black scholars, Ford and Airhihenbuwa, developed the Public Health Critical Race Praxis (PHCRP) in 2010. Prior to 2010, CRT discourses had been lacking in health sciences even when the subject at hand concerned health disparities in relation to race and racism. Ford and Airhihenbuwa (2010) suggest that the lack can be attributed to “CRT’s methodological complexity and jurisprudential orientation,” which differ from public health’s “scientific approach and emphasis on practical application” (p. 1390). The fundamental differences between the two disciplines prompted the authors to develop the PHCRP, a framework that “helps public health researchers to carry out health equity research with fidelity to CRT” (p. 1390).

### Examples of how CRT has been utilized in the field

Since its appearance, researchers have applied PHCRP to their research to address health disparities. Thomas et al. (2011), for example, incorporated PHCRP into their ongoing projects that aim to reduce health disparities. Even though their project had long focused on eliminating health inequities, race and racism were often treated superficially. They believe that PHCRP corrected such flaws by centering race and racism in discussion, addressing the researcher’s own biases, and confronting the power structures that perpetuate inequities.

Also employing PHCRP, the race-conscious epistemology has led health scholars to challenge how race has been conceptualized to explain the causes of endometrial cancer disparities (Doll et al., 2018) and to find that Black and Latinx patients from an academic referral center were less likely to be admitted to cardiology for health failure care (Eberly et al., 2019). Another PHCRP-informed study identified and countered race-related misconceptions in rheumatology (Williams et al., 2020). By “centering the margins” (Ford & Airhihenbuwa, 2010, p. 1391), or relinquishing the White racial frame, these studies reveal how historical injustices and ongoing racism continue to follow people of color in the USA—even through different types of illnesses and treatments.

If racism is normal and not aberrant in the USA, it then should not be a surprise that racism is encountered not only by patients of color but also health professionals of color. Thomas (2018) conducted a CRT-informed study to examine the barriers that International Board Certified Lactation Consultants experienced during the course of their certification. Findings from the research show that for people of color, cost and racial discrimination were identified as primary barriers for certification. Adopting CRT and cognitive load theory, Bullock et al. (2020) examined the prevalence of racial/ethnic-stereotype threat amongst fourth-year medical students in US-based institutions and its impact on students’ clinical experience. Their mixed methods study shows that 82% of Black students had high vulnerability to stereotype threat, followed by 45% Asian, 43% Latinx, and 4% White.

In addition to scholarly research, calls for CRT mushroomed in health education in the latter half of the 2010s. Critically engaging lecture materials on race and racism, or the lack thereof, students and educators from such health fields as psychology (Watkins et al., 2018), medicine (Braun, 2017; Krishnan et al., 2019; Tsai & Crawford-Roberts, 2017), nursing (Valderama-Wallace & Apesoa-Varano, 2020), and pharmacy (Bush et al., 2018; Rockich-Winston, 2018) have sought to reform curricula to address and redress racial injustice that has been embedded therewithin.

### Significance of the field to educators

People are often made to believe that the search for objectivity makes scientists’ work trustworthy, reliable, and without bias. Our literature presents evidence to the contrary. From the conceptualization of race in health education and healthcare access to licensing and practicing, explicit or implicit bias is frequently identified. Higher education institutions play an integral role in transmitting knowledge to next generations, investing in cutting edge medical research and technology, and disseminating health research information to academic and non-academic audiences. Exposing and problematizing the long-held assumptions about race and related practices, CRT challenges the foundational ideology underpinning different institutional endeavors. It is therefore a pressing matter that higher education scholars and practitioners—in healthy fields any beyond—reexamine how the conceptualization of race undergirding research and practices is in need of rectification. With a race-conscious lens and an anti-racist stance, scholars and practitioners may work to include racism and discrimination as determinants of health in research; they may also work to reevaluate policies and presumptions of campus corners that do not usually receive attention, such as student health services, labs, classrooms, or academic medical centers. As inspired students and educators from across health fields call for CRT in health education with greater urgency, it can be expected that more institutions will soon be pressed to revisit curricula and practices to address embedded racism.

### Computer science and information technology

Not unlike health sciences, racism has long been embedded in computer science and information technology (hereafter, computer science). Yet the covert racism that lives on under the guise of progress has been hard to identify. After CRT discourses in computer science mushroomed in the 2010s, scholars have focused on how scientific racism has accompanied the modern-day technological development that ranges from artificial intelligence (AI) and algorithmic systems to facial analysis and social media. Consequently, CRT in computer science unveils another “face” of technology and shows that technology does not appear to be as neutral or harmless as people normally think.

### Examples of how CRT has been utilized in the field

Intending to fill the research gap, Hamilton (2020) delineates a helpful history of critical race and digital studies to provide a contemporary framework for understanding the field. By building on the concept of colorblind racism, a framework pioneered by Eduardo Bonilla-Silva (2002, 2013, 2015), Hamilton’s findings reveal that early tech entrepreneurs and internet researchers adopted colorblind ideals and “actively resisted conversations about race in the structure of the Internet” (p. 293). The fantasy of racial utopia has prevented nuanced discussions of bias and racism in digital technologies, and its impact can still be observed today as indicated by the literature discussed below.

A scholar who has been building upon the discussion about critical race and digital studies is André Brock. Informed by CRT and other perspectives such as feminism and queer theory, Brock (2009) developed critical tchnocultural discourse analysis (CTDA) to examine how race engages with information technology “by situating online discourse about cultural artifacts within a sociocultural matrix” (p. 345). In particular, CTDA incorporates the epistemological standpoints of underserved users in information and communication technologies (ICT) “so as to avoid deficit-based models of underrepresented populations’ technology use” (Brock, 2018, p. 1012). To illustrate CTDA in action, Brock analyzed, for instance, the racial and technological discourses surrounding the release of Blackbird, a browser built on Firefox aimed at African Americans (2011). He found that on technology blogs, colorblind ideology has led many bloggers to believe that the internet is a neutral cultural space, and that the efforts by non-Whites to carve out a space such as Blackbird are not welcome. Through CTDA, Brock attempts to decenter normative discourses where White masculine heterosexuality “overdetermines ICT use and design” (Brock, 2018, p. 1027).

Other topics in computer science which have gained increasing attention in recent years are the Whiteness of AI and how algorithmic systems have reproduced or amplified existing social inequities. In their research, Cave and Dihal (2020) focus on how AI has been predominantly portrayed as White in real or imagined intelligent machines such as human-oid robots, chatbots and virtual assistants, stock images of AI, and portrayals of AI in film and television. Drawing on CRT, the authors offer three interpretations of the Whiteness of AI. First, the normalization of Whiteness has allowed Whiteness to reproduce itself; second, AI is predominantly racialized as White because AI is deemed to possess qualities that are associated with Whiteness including intelligence, professionalism, and power. Third, the Whiteness of the machines permits the White utopian imagination to fully erase people of color. In another work, Scheuerman et al. (2020) examine current approaches in image databases used for facial analysis by synthesizing theory from critical race studies. They conclude that though racial and gender categories throughout these databases are portrayed as objective and apolitical, they may in fact perpetuate harmful and oppressive agendas as there has been a glaring absence of thoughtful engagement with race and gender data which are often shaped by both cultural and historical factors. A case in point is the erasure of trans experiences. The authors suggest facial analysis researchers embrace their positionality and adopt a sociohistorical perspective when making decisions around classifications of race and gender as they situate their databases. Like Brock, these authors draw on CRT among other theories to ground their research as they dismantle dominant ideology that often upholds the White racial frame.

### Significance of the field to educators

Although the discriminatory values at work within computer science are often unnoticed and go unchallenged, our literature shows that technology is in fact not as neutral or harmless as people normally think. Specifically, the literature reveals racism’s ability to alter manifestations and to take shape through different language including programming language.

As the Senate passed bipartisan legislation in June 2021 that would authorize billions of dollars in funding for technology research in the USA (Gravely, 2021), it can be expected that higher education institutions would direct more resources and energies to technological research and development and adopt new technological innovations. The development heightens the urgency for higher education scholars and practitioners to think more critically about the data-driven practices that have swept across US institutions and prized in spheres that range from leadership and student services to student learning and instruction.

A recurring theme that emerged from our literature concerns the White masculine voice that predominates the use and design of information and communication technologies, including deciding how race is conceptualized and operationalized in the field. What thus requires critical examination is the *input* of data-driven practices that involves questions such as: *Who decides the kinds of data to be collected? What is the philosophy underpinning the construction of data sets? What are the historical and cultural contexts of people involved in decision-making? Why are data used in a given way? What are the policies associated with the data and their relation to historical injustices if any?*

As more educators will now play a central role in supporting technology research with increased federal funds, they are urged to look beyond the benefits that technology brings and learn to scrutinize its origins and related ideologies to better tackle scientific racism within the higher education context. Unchecked, algorithms will continue to disadvantage underrepresented minority groups, as shown in the software that some universities use which was claimed to have disproportionately labeled Black and other minority students “high risk” and pushed Black students out of math and science (Feathers, 2021). To redress the colorblind ideology that is as old as the invention of technology itself, educators are called to actively initiate conversations about bias and racism in digital technologies in all levels of meetings to ensure that diverse racial and ethnic voices are properly represented and considered in all decision-making process.

### Sports

Within institutions of higher education, college sports are most often associated with entertainment, donations, drama, and attention: not racism. Due to the large representation of Brown and Black folks in sports, the pervasiveness of racism in the history of sports at both personal and institutional levels is not surprising despite the common belief that robust representation excludes the possibility of discrimination. The easily identified discriminatory values and practices in sports may account for why CRT has gained much traction in sports studies. Countering the public’s proclivity to sidestep the topic of racism in favor of sports entertainment, CRT offers sports studies an epistemic tool to probe behind the façade to reveal how the colorblind logic has been employed in the enterprise. Specifically, CRT challenges sports scholars to make their research political rather than neutral (Hylton, 2010).

### Examples of how CRT has been utilized in the field

Two Black scholars, US-based Singer (2005a, 2005b, 2009) and UK-based Hylton (2005, 2010), were among the first scholars to usher CRT into sports studies. According to Hylton (2010), utilizing CRT in anti-racist sports practice and policy help disrupt the established practices, knowledge, and resources that often lead to colorblindness and racialized processes. He suggests that CRT’s political agenda of challenge is instrumental in empowering sports communities to elevate racial consciousness as they reexamine conceptualizations of race and racialized processes that have long been embedded in theory and practice.

Following Singer and Hylton, sports scholars in the past decade have adopted CRT as an analytical framework to investigate matters around race and racism at various levels. At an empirical level, attention has been given to, for example, the experiences of Black female assistant coaches at predominately White institutions and how they perceive the underrepresentation of Black female head coaches in NCAA Division I women’s basketball (Borland & Bruening, 2010). Adopting a CRT lens, Burden et al. (2005) explore the perceptions of African American faculty in kinesiology-based programs at predominantly White institutions on their organizational socialization in terms of teaching, research, and service. Several CRT-informed works have focused on the impact of race and racism on Black student-athletes (Armstrong & Jennings, 2018; Bimper, 2017; Singer, 2005b). What is worth special mention is the work of Bimper (2017). Guided by CRT, Bimper explores how a student-athlete mentoring program challenged Black student-athletes to critically consider the presence and impact of Whiteness. The program also helped Black student-athletes develop their social capital to become greater self-advocates.

At an institutional level, Bimper and Harrison (2017) draw on CRT to interrogate issues of race and racism that are embedded in organizational policies. They examine the construction of departmental directives and strategic policies in intercollegiate athletic organizations. Their findings show that institutions often uphold colorblind ideology, avoid conversations about persistent racial inequities, and direct organizational energies away from an elevated racial consciousness.

As a growing number of athletes, activists, institutions, and entrepreneurs engage in actions to promote social justice within and beyond sporting spaces, athlete activism has also gained increased attention (e.g., Cooper et al., 2019). Through a CRT lens, Frederick et al. (2019) examine the framing of activist efforts initiated by LeBron James, Dwayne Wade, Chris Paul, and Carmelo Anthony during the 2016 Excellence in Sports Performance Yearly Award. Having analyzed over 10,000 comments made to the three posts on ESPN’s Facebook page pertaining to calls for activism, the authors observed the persistence of cultural stereotypes that are deeply ingrained in US society. They also noted that stereotypical beliefs are often left unshaken by athlete activism.

### Significance of the field to educators

Despite the public’s resistance to the topic of racism, our literature reveals that racism has always been at play in sports at both personal and institutional levels. As college sports has long been and will continue to be an essential part of the college setting, higher education scholars and practitioners would do well in supporting sports programs by means of exposing the long-held colorblind ideology in the field. An immediate action that can be taken is to review the demographics of faculty in kinesiology-based programs, athletics directors, head coaches, and athletic conference commissioners in one’s institutions and conferences and then push for necessary change. Concerned educators are also encouraged to work from the ground up and explore opportunities to assist programming efforts to foster critical consciousness among student-athletes through mentoring and auxiliary services. As sports affords players a unique platform to initiate change, student-athletes—regardless of color—with proper guidance may come to learn how they can use their influence as they participate in the fight for social and racial equality. As they engage in change, educators are to be reminded that whether in research or practices and whether at individual or institutional levels, the nature of the change that CRT presses for—in sports but also other fields—will always be political and not neutral.

### Business

Similar to computer science, CRT is rarely the focus in the broadly defined business field (Harney & Dunne, 2013). Yet adopting a CRT lens, scholars have uncovered how racism has been perpetuated in such entities as the housing market, city planning, finance, and marketing research—in ways that are often unknown to the public. As such, CRT has shown that in the US context, the invisible hand of the market is hardly invisible, but White (Francis & Robertson, 2021).

### Examples of how CRT has been utilized in the field

In business, CRT-informed research has repeatedly shown that the need to sustain White privilege and protect White spaces is what leads to the discriminatory practices that create and perpetuate inequities for communities of color. Drawing on CRT and interdisciplinary research, Francis and Robertson (2021) examine ways in which real estate agents, lenders, and retailers engage in racially discriminatory practices—often covert these days—which lead to marketplace inequities for Black consumers. An example is a strategy called block-busting that is used by real estate agents to scare White homeowners into selling by insinuating that the newly arrived Black residents could potentially lead to increased crime and decreased property values in the neighborhood. In another study, with a CRT lens, Safransky (2020) examines an algorithmically produced market value assessment that is used to guide development in cities in such a way as to disadvantage racialized others across the US. Safransky argues that data-driven analytics are neither neutral nor objective but that the algorithmic violence associated with data production can potentially lead to a new kind of municipal redlining and thus needs to be problematized.

Informed by CRT, Lewis (2015) discusses the underrepresentation of Black certified public accountants in the USA and the challenges of Black professionals to maintain a successful career in the field. In addition to navigating ongoing microaggression, Black professionals need to constantly manage their identity that is seen as “deficit” and rework it into valuable capital so that they may better fit into the “white institutional ideal demanded by the Whiteworld of accountancy” (p. 10). In these cases, White spaces and White normativity are considered an entity that should be safeguarded but also emulated.

It is likely that the racially discriminatory practices in the business industry are simply a reflection of the spaces that generate and impart racially discriminatory values. Drawing upon CRT, Dar et al. (2020) forcefully critique the business schools and scholars in the Global North/West for universalizing their experiences, upholding White supremacy by perpetuating a racist logic, and undermining Global South experiences and knowledge. Poignantly, the authors point out that some of the efforts surrounding diversity, equity, and inclusion in business schools stay at a superficial level and never challenged White power. They call for collective action to build a movement for anti-racist scholar-activism and urge scholars involved to use their scholarship to ground their activism. Also, within business schools, Grier and Poole (2020) use CRT as an analytic framework to explore the experiences of underrepresented minority faculty who have served on business school search committees. Among several themes that emerged from interview participants’ narratives include tokenization (i.e., the same faculty of color are repeatedly asked to serve on the search committee to bring the racial perspective) and a racialized faculty search process.

Engaging CRT extensively in their work, Poole et al. (2021) discuss the contributions of CRT in marketing research. For instance, CRT challenges mainstream approaches in marketing research that often center on how the behaviors and attitudes of people of color “deviate” from dominant social norms. Also, when addressing marketplace racism, CRT shifts the blames from individual consumers to power structures, institutional norms, and policies that are racially oppressive. The authors believe that adopting a CRT lens in marketing research will help us understand consumers’ lived experiences in a more nuanced way, which in turn will lead to a more equitable version of the marketplace.

### Significance of the field to educators

In an age where the commodification and marketization of education have become a reality, talking about CRT and racism when dollars are on the line seems inappropriate and an imposition. Yet, the resistance may be even more reason that disrupting the status quo is necessary because without it, higher education institutions run the risk of perpetuating racial disparities through domains that seldom allowed critical inspection.

Though combating the oppressive marketing structures that uphold White power can be a daunting task, our review provides several strategies that higher education scholars and practitioners may take to initiate change. As CRT takes seriously the lived experiences of people of color, scholars and practitioners are urged to call out marketing strategies, processes, and products that omit people of color’s presence or characterize them from a deficit view. Additionally, the practice of tokenism that only makes a symbolic effort concerning diversity and inclusivity is to be interrogated so as to push for full integration of underrepresented employees in the higher education workplace at all levels. Also, requiring scrutiny is the “minority tax,” or the extra responsibilities placed on underrepresented faculty in the name of diversity. Concerned scholars and practitioners are urged to reexamine the salary review process to better account for this additional workload. As with all other fields discussed in this paper, racism in business is often hidden behind technical terms. To identify inequities and related ideologies and press for change, it behooves higher education scholars and practitioners to engage research, lecture materials, advertising strategies, and budget and financial reports with a critical eye and thoughtful queries.

### Religious studies

Unlike the previous four fields, religious studies are often sidelined in non-faith-based institutions of higher education. Still, CRT has made inroads into the field to interrogate how racism—often by joining forces with power structures—has manifested through religious realms. That more than 70% of US adults are religiously affiliated (Pew Research Center, 2019) makes the inclusion of this subsection imperative.

### Examples of how CRT has been utilized in the field

In religious studies, CRT “seeks to understand the social constructions of race as they have changed over time, determining what paradigms get created and how they replicate themselves and operate in complex dynamics of power” (Brettschneider, 2015, p. 108). In other words, religious scholars who engage CRT investigate who “wins” and who “loses” in power structures as well as how humans have been categorized in accordance with contemporary political ideas. Adopting a Jewish critical race theory lens that privileges matters of Jewish import in analyses, Brettschneider (2015) dialogues with two books on Black Jews in Africa in her work: *Black Jews in Africa and the Americas* (2013) by Tudor Parfitt and *The Black Jews of Africa: History, Religion, Identity* (2008) by Edith Bruder. While affirming their contributions, Brettschneider challenges the books for their centrality of Whiteness, their reinforcement of a Black-White dichotomy, and their lack of theoretical or theological space for Jews of color. In addition, Brettschneider notes that stories from Africa are often validated only if they correspond to stories in the Bible and she questions such line of thought.

In addition to Jewish studies, CRT has also been adopted to examine anti-Muslim sentiment as well as the perception of racial inequalities among White conservative Protestants in the USA. Drawing on CRT and critical Whiteness studies, the research of Tranby and Hartmann (2008) shows that compared to other Americans, White conservative Protestants are less aware of White privilege and that they are less likely to perceive the structural barriers facing African Americans. Further, White conservative Protestants were found to be more likely than other White Americans to believe that their race is very important to their identity. Such findings suggest that for conservative Protestants at least, Whiteness is not a “hidden” identity as some people claim but a “very visible and real thing” (p. 353). The findings also provide grounds for further research on the implications of differing perspectives of Whiteness.

Guided by CRT and the study of cultural boundaries in national belonging, Gerteis et al. (2020) explore the anti-Muslim sentiment in the USA. They found that nearly half of Americans embrace some form of anti-Muslim sentiment, and that “the more Americans embrace colorblind liberalism and reject racial nationalism, the less likely they are to reject the idea that Muslims belong in the public sphere” (p. 730). Understanding their findings within broader discourses of belonging, Gerteis et al. conclude that anti-Muslim sentiment is not simply a religious or racial form of prejudice. To better understand such sentiment requires one to also consider discourses around civic life, American identity, and the assumed cultural bases of citizenship.

Even though it is often believed that religion is where one’s full humanity is found, history is, unfortunately, filled with racist incidents, often in the name of religion. To account for the phenomenon, Heschel (2015), informed by CRT, explores the appeal of racism through a religious and ethical perspective. For her, the “slippery yet tenacious” nature of racism “facilitates the racialization of both religious thought and social institution” (p. 3). Consequently, racism is hard to define and often unrecognized. Even as we repudiate racism, says Heschel, humans may still perpetuate it unwittingly. She concludes that the roots of racism’s tenacity need to be sought “not simply through their outward manifestations but also in our deepest, most hidden, and even unconscious motivations” (p. 23). Heschel’s study reminds us that all humans are held accountable to the dehumanizing acts in which we have and continue to participate.

### Significance of the field to educators

Since religion has become a private, personal matter in US society because of the separation of church and state, spirituality has often been squashed in the public square. Being a microcosm of US society, similar patterns are observed on college campuses where spirituality is believed to be a private affair which does not deserve attention from higher education scholars and practitioners. A CRT-guided philosophy would argue otherwise. Because people who are religiously affiliated seldom consider how racism is manifested through religious realms, higher education institutions are well-positioned to initiate and facilitate conversations that challenge people’s presuppositions of religious beliefs. By better informing the public of racism’s ability to hide even behind the most seemingly sacred spheres or professions, we can hope that less harm will be done in the name of religion. Developing the ability to think more critically about racism in relation to religious institutions, I believe, will help us practice the love and justice that each religion teaches more authentically.

## Conclusion and implications

Traditionally, CRT in higher education has been used to analyze and critique educational research and practice, with a focus on such areas as colorblindness, academic curriculum, campus climate, and affirmative action. By showing that CRT is also germane to health sciences, computer science, sports, business, and religion—fields where racism has long existed but rarely acknowledged let alone named—this study argues that a more expensive look at CRT in higher education is not only urgently needed but will also become inevitable in future research. It is critically important for educators to identify colorblind ideology underpinning research and practices while orienting their work around diversity, equity, and inclusion through a CRT lens. But beyond that, this paper calls for an expansion of the application and scope of research and practice when engaging CRT in higher education given that structural inequality requires a broad, structural reform. Put differently, to more comprehensively address structural racism within institutional bodies, educators would do well by also considering ways in which the different domains of higher education institutions—as illustrated in this paper—are implicated while researching on, for instance, campus climate or affirmative action. The complexity of higher education administrative structures warrants scholars and practitioners from across disciplines and departments to rely on one another’s expertise and collaborate creatively so as to expose and dismantle the racist ideologies at work underneath the technicalities in corners that have so far eschewed examination.

Instead of claiming expertise in all fields discussed, it was this paper’s design to provide only examples of research to illustrate how scholars have utilized CRT in their fields and showcase the breadth of the application of CRT in less-expected higher education spheres. Due to the limited scope of this paper, only selected areas were included, and only initial recommendations on anti-racist policies and practices relating to each field were offered. Readers are therefore encouraged to refer to the literature in each field to explore the subject matter in more depth and consider action points that have not been covered in this work. Other areas of higher education which were left out but merit close examination due to their lesser-known presence within the overall CRT corpus include STEM education, media, and queer studies, to name a few. Future scholars are encouraged to investigate these areas in their research endeavors to uncover how race has been conceptualized and operationalized in these fields. Based on these studies, future scholars may also engage in interdisciplinary or multidisciplinary inquiries as they develop a more comprehensive planning framework that targets structural racism within higher education institutions. Last but not least, as CRT has become an eye-catching term, co-opting the language of CRT for researchers’ own purposes may become a common tactic that requires careful scrutiny to ensure that the potency of CRT is not compromised.
